# Association of preeclampsia with anthropometric measures and blood pressure in Indian children

**DOI:** 10.1371/journal.pone.0231989

**Published:** 2020-05-05

**Authors:** Karuna Randhir, Hemlata Pisal, Vrushali Kadam, Amrita Khaire-Ghadge, Nandini Malshe, Ruma Deshpande, Sonali Palkar, Sanjay Lalwani, Kalyanaraman Kumaran, Chittaranjan Yajnik, Clive Osmond, Caroline Fall, Sadhana Joshi

**Affiliations:** 1 Mother and Child Health, Interactive Research School for Health Affairs, Bharati Vidyapeeth (Deemed to be) University, Katraj, Pune, India; 2 Dept. of Pediatrics, Bharati Hospital and Research Centre, Bharati Vidyapeeth (Deemed to be) University, Katraj, Pune, India; 3 MRC Lifecourse Epidemiology Unit, University of Southampton, Southampton, United Kingdom; 4 Diabetes Unit of KEM Hospital and Research Centre, Rasta Peth, Pune, India; University of Cambridge, UNITED KINGDOM

## Abstract

**Background and objective:**

Birth weight and post-natal growth are important predictors of adult health. Preeclampsia (PE) is associated with low birth weight and may have long term effects on the health of the children. The current study aims to compare anthropometry and blood pressure between children of mothers with and without PE in an Indian cohort.

**Methods:**

We studied children born to women with (PE; n = 211) and without preeclampsia (non-PE; n = 470) at Bharati Hospital, Pune, India. Anthropometry and blood pressure were measured in children at 3–7 years of age. Weight and height Z-scores were calculated using the WHO 2006 growth reference. Independent t-tests were used to compare means between the two groups, and associations between preeclampsia and child outcomes were analyzed using multiple linear regression, adjusting for potential confounders.

**Results:**

Weight and height Z-scores (p = 0.04 and 0.008), and subscapular skinfold thickness (p = 0.03) were higher among children of PE compared with children of non-PE mothers. Systolic blood pressure was also higher in children of PE mothers (1.70 mmHg [95% CI 0.05, 2.90] p = 0.006). BMI and diastolic blood pressure did not differ between groups. In regression models adjusted for newborn weight and gestational age, current age and sex, and maternal height, BMI and socio-economic status, children of PE mothers had higher weight Z-score (0.27 SD [95%CI 0.06, 0.48] p = 0.01), height Z-score (0.28 SD [95%CI 0.09, 0.47] p = 0.005), and subscapular skinfold thickness (0.38 mm [95%CI 0.00, 0.76] p = 0.049). A trend for higher systolic blood pressure (1.59 mmHg [95%CI -0.02, 3.20] p = 0.053) in the children was also observed in the adjusted model. The difference in systolic blood pressure was attenuated after adjusting further for the child’s weight and height (1.09 mmHg [95%CI -0.48, 2.67] p = 0.17). There was no evidence of differences in effects between boys and girls.

**Conclusion:**

Children of PE mothers were taller and heavier, and had higher systolic blood pressure, partly explained by their increased body size, than children of non-PE mothers. *In utero* exposure to preeclampsia may increase the risk of future cardiovascular disease.

## Introduction

Cardiovascular disease (CVD) is the leading cause of mortality globally and imposes a heavy burden on health care systems [1,2]. Research linking lower birth weight to an increased risk of CVD in adult life [3] has led to the theory that an adverse environment during fetal development leaves a lifelong susceptibility to CVD, a concept known as the ‘developmental origins of health and disease (DOHaD)’ [4,5] Preeclampsia (PE) is a common complication of pregnancy, characterized by maternal hypertension and proteinuria, and altered placental blood vessel development which reduces blood flow and nutrient delivery to the fetus, leading to fetal growth restriction [6,7]. PE is also associated with an increased risk of pre-term birth, another cause of reduced birth weight.

Previous studies in high income countries (HICs), have investigated the effects of maternal PE on height, weight, body mass index (BMI), other measures of adiposity, and blood pressure among children or adults. These have shown that preeclampsia is associated with higher weight and body mass index in adolescents and adults [8,9,10, 11, 12, 13]. Taller height or accelerated childhood height gain have also been reported, though less consistently [8,14]. Several studies and two systematic reviews [10,15] have also found that offspring born to mothers with preeclampsia exhibit higher systolic and diastolic blood pressure as children, adolescents and adults [9,11,12,16,17,18,19,20,21]. Sex specific changes, and differences in effects depending on whether preeclampsia was mild, moderate or severe, have been described in some of these studies.

Greater childhood adiposity and higher childhood blood pressure are known to be associated with an increased risk of obesity and hypertension respectively in adult life, and thus children of women with preeclampsia may be at increased risk of cardiovascular disease [22]. Intra-uterine growth restriction [23] and PE [24] are more common in low- and middle-income countries (LMICs) than in HICs (the prevalence of PE in India is estimated at 8–10%) [25]. The incidence of cardiovascular disease is rising in LMICs; deaths from cardiovascular disease have overtaken those due to infectious disease in India and are higher per unit population than in HICs [26].

To the best of our knowledge, there are no published follow-up data from children born to mothers with preeclampsia in LMICs. Previous studies carried out in our department have demonstrated higher maternal homocysteine, oxidative stress, altered placental angiogenesis and disturbed fatty acid metabolism in women with preeclampsia [27,28,29]. We have also reported altered placental global and gene specific methylation patterns in angiogenic factors in preeclampsia [30]. These changes could have implications for early life programming of later life cardiovascular disease. The current study followed up a cohort of children born to women with and without preeclampsia and compared their anthropometric measures and blood pressure at age 3–7 years in an Indian cohort.

## Methods

### Recruitment of mothers and newborns at delivery

The study was carried out at the Interactive Research School for Health Affairs (IRSHA), in the city of Pune, India, in collaboration with the departments of Obstetrics and Gynaecology and Pediatrics, Bharati Hospital, Bharati Vidyapeeth University, Pune, India. The mothers were recruited on admission for delivery during 2006 to 2015. The study was initially set up to study maternal and cord blood bio-markers in relation to obstetric/birth outcomes, especially preeclampsia, pre-term birth and low birth weight [27,31,32,33].

Obstetric staff explained the study to all women admitted to the obstetric unit in labour and recruited women willing to participate. Inclusion criteria were: maternal age 18–35 years and married. Exclusion criteria were: a) a history prior to this pregnancy of chronic non-communicable disease (diabetes, kidney failure, hypertension and CVD), b) a history of gestational diabetes diagnosed during this pregnancy; c) alcohol or substance abuse (not including tobacco use), d) multiple pregnancy (twins/triplets), or e) known HIV or HBsAg (hepatitis B Australia antigen) positivity. Recruitment was suspended at times when the unit was under severe pressure and staff were too busy with clinical tasks. At recruitment, maternal age, parity, and pre-delivery weight and height were recorded. Mothers were asked if they used tobacco or consumed alcohol. We recruited women having preeclampsia irrespective of whether they had a history of hypertension in an earlier pregnancy. Similarly, women without preeclampsia were assigned as controls regardless of their history in previous pregnancies.

After delivery, the pregnancies were classified into 2 groups: 1) a preeclampsia group (‘PE’) of women diagnosed by the obstetrician to have preeclampsia at any stage of pregnancy, based on 1+ proteinuria on dipstick testing (equivalent to 300mg/24 hours or more) and high blood pressure, defined as systolic BP >140 mmHg and/or diastolic BP >90 mmHg; and 2) a control group of women with no evidence of preeclampsia at any stage of pregnancy (‘non-PE’) and comprising women with uncomplicated pregnancies with gestation >37 weeks and birth weight >2500 g, women with pre-term deliveries (<37 weeks completed gestation), and women whose newborns were of low birth weight (birth weight <2500g at gestation 37 weeks or more.

### Follow-up of children at 3–7 years of age

**Fig 1:**
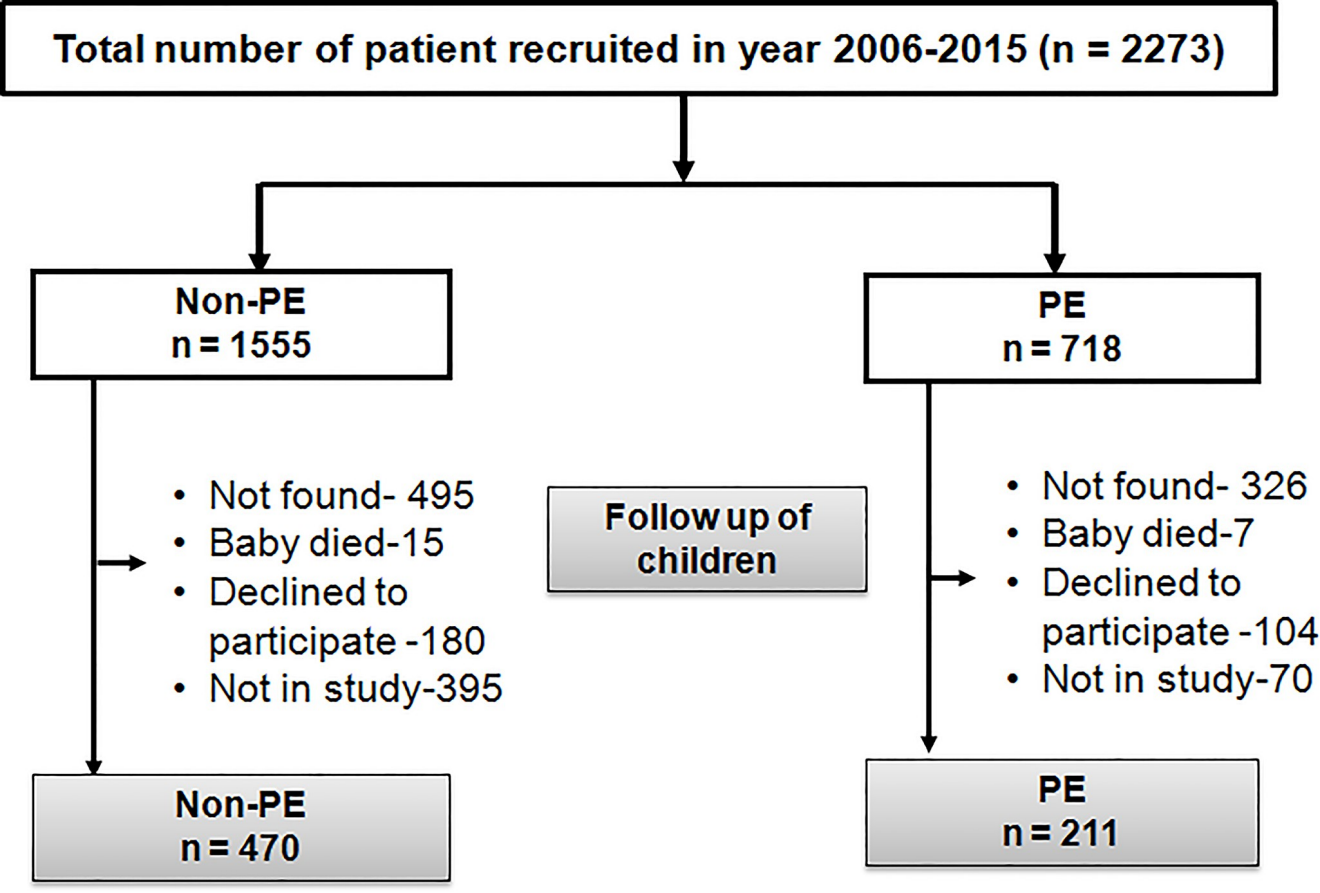
Numbers of births in each group in the original recruited cohort and the numbers studied at the age of 3–7 yr.

There was no contact with the families after birth until the 3–7 year follow-up, which was carried out between July 2013 and October 2017. Contact phone numbers and addresses were obtained from hospital records. Families were contacted by telephone, or by home visit if the phone number was missing or no longer valid. Some children (821 [36%]) were untraceable because their families had moved away from the recorded address (‘Not found’ in Fig 1); 22 [1%] had died, and 284 [12%] did not want to participate in the current study. Numbers of births in each group in the original recruited cohort and the numbers studied at the age of 3–7 years are shown in Fig 1. Some children were traced but not studied because the funding was time-limited, and they could not be studied before funding finished (‘Not in Study’ in Fig 1).

### Investigations at 3–7 years of age

#### Socio-demographic data

We collected details of the mother’s educational level, occupation and current socio-economic status (SES) using the Standard of Living Index (SLI), a standardised questionnaire designed by National Family Health Survey-2 [34]. The SLI questionnaire records information regarding the type of house, water and sanitation facilities, land ownership and household possessions, and produces a composite score; a higher score indicates higher socio-economic status.

#### Anthropometry

The children’s anthropometry was measured by one of three trained project assistants. Weight was measured to the nearest 0.1 kg using a Tanita HD-382 Digital weighing scale. Standing and sitting height were measured to the nearest mm using a Seca 206 Stadiometer, with the head held in the Frankfort plane. Triceps, biceps, subscapular and supra-iliac skinfolds were measured to the nearest millimeter using Harpenden skinfold callipers (John Bull, British Indicators). Circumferences were measured using non-stretchable anthropometric tapes, including occipito-frontal head circumference and mid-upper arm (halfway between the acromial and olecranon processes with the arm flexed). Blood pressure was measured using a sphygmomanometer (Omron 705IT) after at least 5 minutes sitting at rest. The child’s mid-upper-arm circumference (MUAC) was measured and the correct cuff size was chosen. Three readings were recorded at intervals of 5 minutes, and the average used for analysis. Mothers accompanied their children at the time of measurement and the children were comfortable (often sitting on the mother’s lap) between measurements. Data collection staff were kept strictly blind to the mother’s preeclampsia status.

Ethical approval for the original study and the children’s follow-up study was obtained from the Bharati Vidyapeeth Institutional Ethics committee (BVDU/MC/13; BVDU/MC/52). Mothers gave individual informed written consent at the time of recruitment, and either the mother or father gave informed written consent at the time of the child’s follow-up.

### Statistical methods

Data are presented in tables as means (SD), medians (IQR) or Ns (%) for normally distributed continuous variables, continuous variables with a skewed distribution and categorical variables respectively. Maternal and newborn characteristics at the time of delivery, and outcomes at 3–7 years, were compared between PE and non-PE groups, using t tests for normally distributed continuous variables and Chi square tests for categorical variables. For these comparisons, the child outcomes were adjusted for age and sex. Anthropometric outcomes were also converted into age- and sex-specific Z-scores using the WHO 2006 reference (WHO Multicentre Growth Reference Study Group, 2006) and compared between groups. Associations between the exposure of interest (PE/non-PE as a binary variable) and child outcomes were then analyzed using multiple regression. We initially examined the univariate associations of outcomes (BP and anthropometry) with potential confounders: birthweight, gestational age and maternal BMI, height and socio-economic status (S1 Table). There were significant associations of birth weight, gestational age, maternal size and SES with the child’s anthropometric measures, though not with BP. Given that there were significant differences between PE and non-PE groups in birth size, gestational age and maternal characteristics, we decided it would be best to take a uniform approach for all outcomes and carried out a series of three models for all as follows: 1) adjusted for child age and sex; 2) additionally adjusted for birth weight and gestational age at birth; and 3) additionally adjusted for maternal SLI score, height and BMI.

Skewed variables (SLI scores) were transformed to normality for use in regression models. Because several studies have reported different associations of maternal preeclampsia with child outcomes between boys and girls, we repeated these regression analyses stratified by child sex, and conducted interaction tests. Finally, we examined the representativeness of the study sample by comparing maternal and newborn characteristics of the children included in our study and the remainder of the original cohort of births.

## Results

### Maternal characteristics

The weight and BMI of women with PE were higher than women in the non-PE group (Table 1). As expected, maternal blood pressure was higher among PE women, while newborn gestational age, placental weight and all newborn measurements were lower. Maternal education level and SLI scores were similar in both groups.

**Table 1:** Comparison of maternal and newborn characteristics between PE and non-PE groups.

	N	Non-PE	n	PE	p
**Maternal characteristics**					
Age (yr)	470	23.7 ± 3.5	211	24.1 ± 3.9	0.28
Height (cm)	462	152.7 ± 5.8	186	153.6 ± 6.1	0.09
Weight (kg)	365	57.4 ± 9.3	171	64.0 ± 11.8	0.001
BMI (kg /m^2^)	362	24.5 ± 3.7	161	27.1 ± 4.7	0.001
Gestational age (wk)	470	38.9 ± 1.7	211	37.5 ± 2.6	0.001
Systolic Blood Pressure (mmHg)	470	122.4 ± 8.5	211	147.5 ± 15.6	0.001
Diastolic Blood Pressure (mmHg)	470	78.2 ± 5.2	211	96.1 ± 10.8	0.001
SLI score	423	30 (26,32)	198	30 (28,32)	0.16
Maternal education		N (%)		N (%)	
Post graduate		27 (5.7)		17 (8.1)	
Graduate		81 (17.2)		44 (20.9)	
Higher secondary		99 (21.1)		35 (16.6)	
Secondary		236 (50.2)		104 (49.3)	0.64
Primary		13 (2.8)		5 (2.4)	
Vocational		1 (0.2)		1 (0.5)	
Illiterate		13 (2.8)		5 (2.4)	
Placental weight (gm)	279	497.3 ± 105.6	144	468.0 ± 147.6	0.02
**Newborn characteristics**					
Weight(gm)	470	2776.2 ± 405.0	211	2434.3 ± 669.9	0.001
Length (cms)	455	47.9 ± 3.1	202	46.4 ± 4.1	0.001
Head circumference (cm)	457	33.8 ± 1.8	203	32.3 ± 2.3	0.001

**Values are expressed as mean** ± **SD or n (%); the skewed variable (SLI score) is expressed as median (IQR)**

### Child outcomes

The children’s anthropometric and blood pressure outcomes are shown in Table 2. Weight and height, and their Z scores, and subscapular skinfold thickness, were higher among children born to PE compared with non-PE mothers. Systolic blood pressure was higher in children of PE mothers (1.70 mmHg [95% CI 0.05, 2.90] p = 0.006) while there was no significant difference in diastolic blood pressure.

**Table 2:** Child outcomes at age 3–7 years.

	n	NON-PE	n	PE	P
**Anthropometry**					
Weight (kg)	469	16.1± 2.4	205	16.6 ± 3.1	0.03
Z score weight	469	-1.4 ± 1.0	205	-1.2 ± 1.3	0.04
Height (cm)	469	107.6 ± 5.0	205	108.5 ± 5.3	0.06
Z score height	469	-0.9 ± 1.0	205	-0.7 ± 1.1	0.008
Body mass index (BMI, kg/m^2^)	469	13.8 ± 1.2	205	13.9 ± 1.7	0.24
Z score BMI	469	-1.3 ± 1.0	205	-1.2 ± 1.3	0.62
Mid upper arm circumference (cm)	467	15.5 ± 1.3	202	15.7 ± 1.6	0.12
Head circumference (cm)	468	48.3 ± 1.3	202	48.2 ± 1.4	0.23
Triceps skinfold (mm)	466	7.2 ± 1.7	200	7.4 ± 2.2	0.30
Biceps skinfold (mm)	467	5.5 ± 1.4	201	5.8 ± 1.9	0.09
Subscapular skinfold (mm)	465	5.6 ± 1.4	200	6.0 ± 2.3	0.03
Suprailiac skinfold (mm)	462	7.5 ± 2.8	198	8.0 ± 3.4	0.08
**Blood pressure**					
Systolic blood pressure (mmHg)	420	95.0 ± 6.8	194	96.6 ± 7.1	0.006
Diastolic blood pressure (mmHg)	420	60.7 ± 6.8	194	61.2 ± 7.1	0.40

**Except for Z-scores, mean values were adjusted for the age and sex of the child**

The regression analysis is shown in Table 4. Although sex-stratified regression analysis suggested that the association of maternal PE with greater weight and height in the children was stronger in boys than girls (S2 Table) none of the interactions were statistically significant; Table 3 therefore presents data with the sexes pooled. The findings largely mirrored the results shown in Table 2 and were little changed after adjusting for the child’s birth weight and gestational age at birth and maternal anthropometry and SLI score. In the fully-adjusted model, weight, height and BMI Z scores were (0.27 SD [95% CI 0.06, 0.48] p = 0.01), (0.28 SD [95% CI 0.09, 0.47] p = 0.005) and (0.13 SD [95% CI -0.09, 0.36] p = 0.25) higher in the PE group (Table 3). Children born to mothers with preeclampsia also had larger subscapular skinfold thickness. There was a trend for increased systolic blood pressure in PE children (1.59 mmHg [95% CI -0.02, 3.20] p = 0.053). After further adjustment for the children’s own weight and height, the equivalent figures were (1.09 mmHg [95% CI -0.48, 2.67] P = 0.17). Diastolic blood pressure was higher in children of PE mothers though this was not statistically significant in any of the models.

**Table 3:** Multiple regression analysis: Associations of maternal PE with anthropometric and blood pressure outcomes in the children.

	**Unadjusted**			**Adjusted for birth weight and gestational age**			**Adjusted for birth weight, gestational age and maternal BMI, maternal height and SLI score**		
	n	Regression coefficient B (95% CI)	p	n	Regression coefficient B (95% CI)	p	n	Regression coefficient B (95% CI)	p
**Anthropometry**									
**Z score weight**	673	0.19 (0.01, 0.37)	0.04	673	0.41 (0.23, 0.59)	0.001	467	0.21 (0.059, 0.48)	0.01
**Z score height**	673	0.23 (0.06, 0.39)	0.008	673	0.39 (0.22, 0.55)	0.001	467	0.28 (0.09, 0.47)	0.005
**Z score BMI**	673	0.05 (-0.13, 0.23)	0.58	673	0.22 (0.04, 0.40)	0.016	467	0.13 (-0.09, 0.36)	0.25
	**Adjusted for child age and sex**			**Adjusted for child age and sex, birth weight and gestational age at birth**			**Adjusted for child age and sex, birth weight, gestational age and maternal BMI, maternal height and SLI score**		
	n	Regression coefficient B (95% CI)	P	N	Regression coefficient B (95% CI)	p	N	Regression coefficient B (95% CI)	p
**Mid-upper-arm circumference (cm)**	668	0.19 (-0.04, 0.42)	0.11	668	0.38 (0.14, 0.62)	0.002	464	0.23 (-0.06, 0.52)	0.12
**Head circumference(cm)**	669	-0.14 (-0.36, 0.09)	0.23	669	0.12 (-0.11, 0.34)	0.32	465	0.12 (-0.16, 0.40)	0.40
**Triceps skinfold (mm)**	665	0.17 (-0.15, 0.48)	0.30	665	0.38 (0.06, 0.71)	0.02	462	0.16 (-0.25, 0.57)	0.45
**Biceps skinfold (mm)**	667	0.23 (-0.03, 0.48)	0.08	667	0.35 (0.08, 0.62)	0.012	463	0.21 (-0.13, 0.54)	0.23
**Subscapular skinfold (mm)**	664	0.32 (0.04, 0.60)	0.03	664	0.44 (0.15, 0.74)	0.003	461	0.38 (0.002, 0.77)	0.049
**Suprailiac skinfold (mm)**	659	0.45 (-0.05, 0.96)	0.08	659	0.46 (-0.07, 0.10)	0.09	456	0.42 (-0.24, 1.09)	0.21
**Blood pressure**									
**Systolic BP (mmHg)**	613	1.70 (0.50, 2.90)	0.006	613	1.71 (0.45, 2.98)	0.008	446	1.59 (-0.02, 3.20)	0.053
**Diastolic BP (mmHg)**	613	0.52 (-0.67, 1.72)	0.39	613	0.65 (-0.62, 1.91)	0.32	446	0.78 (-0.82, 2.37)	0.34

### Representativeness of the study sample

Table 4 shows data comparing the mothers and newborns in our study sample (the children successfully re-traced and studied at the age of 3–7 years) with the remainder of the original cohort of births in this study. Maternal weight and BMI, and all newborn measurements were smaller in the not-studied groups, both in the PE and non-PE categories.

**Table 4:** Comparison of maternal and birth characteristics between children studied at age 3–7 years and the remainder of the original births.

**Non-PE group**	**n**	**Studied**	**n**	**Not studied**	**p**
**Maternal**					
Age (years)	470	23.7 ± 3.5	1081	23.4 ± 3.5	0.12
Weight at delivery (kg)	365	57.4 ± 9.3	894	55.5 ± 9.3	0.001
Height (cm)	462	152.7 ± 5.8	978	152.7 ± 6.0	0.94
BMI (kg/m^2^)	362	24.5 ± 3.7	856	23.7 ± 3.6	0.25
Systolic blood pressure (mmHg)	470	122.4 ± 8.5	1077	121.8 ± 8.8	0.22
Diastolic blood pressure (mmHg)	470	78.2 ± 5.2	1077	77.7 ± 5.6	0.10
**Newborn**					
Weight (kg)	470	2776.2 ± 405.0	1082	2547.5 ± 519.4	0.001
Length (cm)	455	47.9 ± 3.1	1051	46.99 ± 3.6	0.001
Head circumference (cms)	457	33.8 ± 1.8	1050	33.02 ± 2.4	0.001
Placental weight (gm)	279	497.3 ± 105.6	833	458.43 ± 115.5	0.001
Gestation (wks)	470	38.9 ± 1.7	1078	37.8 ± 2.7	0.001
**PE group**					
**Maternal**	**n**	**Studied**	**n**	**Not studied**	**p**
Age (years)	211	24.1 ± 3.9	506	24.2 ± 4.1	0.72
Weight at delivery (kg)	171	64.0 ± 11.8	429	61.1 ± 11.3	0.006
Height (cm)	186	153.6 ± 6.1	429	152.7 ± 5.9	0.08
BMI (kg/m^2^)	161	27.1 ± 4.7	376	26.1 ± 4.5	0.03
Systolic blood pressure (mmHg)	211	147.5 ± 15.6	506	146.2 ± 17.3	0.32
Diastolic blood pressure (mmHg)	211	96.1 ± 10.8	506	95.5 ± 11.5	0.51
**Newborn**					
Weight(gm)	211	2434.3 ± 669.9	502	2324.4 ± 668.7	0.046
Length(cm)	202	46.4 ± 4.1	489	45.8 ± 4.1	0.059
Head circumference (cm)	203	32.3 ± 2.3	490	32.1 ± 2.7	0.45
Placenta weight(gm)	489	468.0 ± 147.6	415	445.4 ± 121.9	0.07
Gestation (wks)	211	37.5 ± 2.6	503	37.1 ± 2.9	0.14

## Discussion

### Summary of findings

In this first report of child outcomes in relation to maternal preeclampsia from a low-/middle-income country, we found differences in anthropometry and blood pressure in children born to Indian mothers who had preeclampsia compared with children of mothers without preeclampsia. After adjusting for potential confounders, children of PE mothers were taller, heavier and more adipose (based on subscapular skinfold thickness) and had higher systolic blood pressure at 3–7 years of age. Blood pressures in both groups were similar after adjusting for the children’s weight and height.

### Anthropometry

Newborn measurements in our study were smaller in babies of mothers with PE, consistent with the known associations of PE with intra-uterine growth restriction and poorer birth outcomes. However, at 3–7 years of age, we observed higher weight, height, and subscapular skinfold thickness in these children, independent of maternal size and socio-economic status. Thus, children of PE mothers not only ‘caught up’ with the children of non-PE mothers, but also overtook them. Several of the studies already mentioned have reported that exposure to preeclampsia *in utero* is associated with higher weight and/or BMI in adolescents [8,11,12, 13, 20] and adults.^9^ The systematic review by Davis et al. showed, in a meta-analysis of data from seven studies of children, adolescents or adults, that BMI was higher by 0.62 kg/m^2^ (95% CI 0.41, 0.84). Greater adiposity, assessed using skinfolds, has been reported in children of PE mothers [35]. Taller height or accelerated childhood height gain have also been reported in some studies [8,14]. The mechanisms responsible for not only catch-up in weight, height and adiposity compared with children of normotensive mothers, but also an ‘overshoot’, are unknown, but, may result from programmed changes in growth factors as a result of PE exposure. Catch-up growth is regulated by several factors; genetic and environmental factors, and biological regulators such as the growth hormone-insulin growth factor (IGF) system. A recent report from India demonstrated that infants born small for gestational age (SGA) show catch-up growth (both weight and length) at 18 months and this was correlated with higher IGF-1 levels [36]. A longitudinal study from Chile also reported similar findings in SGA babies at 3 yrs of age [37]. However, no studies have examined the role of the IGF system in the regulation of post natal growth in children of PE mothers.

It is important to note that some previous studies have found no effect of PE exposure on children’s weight or BMI [38, 39] or height [11, 38, 39]. A recent study reported findings in the opposite direction for adiposity: lower rather than higher body fat percentage and smaller skinfolds in children born to mothers with preeclampsia and gestational hypertension at 8 years of age [18]. It was interesting in our study that, despite increased weight and skinfolds, children’s BMI was not significantly different between PE and non-PE groups and may possibly be because both height and weight are higher. Alternatively it may be related to body composition; Indians tend to have a lower lean body mass, and at a given BMI a higher fat mass and central body fat, than white Caucasians [40]. Other discrepancies across studies may be due to different study designs, differences in clinical management of PE and other hypertensive disorders in pregnancy, and ages of assessment of the children. In the Davis et al. systematic review, the increase in BMI in offspring of PE mothers was clear only in adolescence or older [10]. Future studies following up the offspring of PE mothers should include better measures of body composition, such as dual-X-ray absorptiometry, to determine whether differences in weight are due to changes in lean or fat mass.

### Blood pressure

The regression analysis revealed a higher systolic blood pressure (by 1.70 mmHg [95% CI 0.05, 2.90] p = 0.006) in children of mothers with PE, a difference that was of borderline significance after adjusting for the child’s birth weight, gestational age at birth, current age and sex, and maternal size and socio-economic status. These results are consistent with other studies in HIC settings, which have shown that systolic blood pressure is higher by ~1–4 mmHg in child, adolescent or adult offspring of mothers with PE or other hypertensive disorders in pregnancy [9, 11,12,13, 17, 18, 20, 21]. Most of these studies also showed higher diastolic blood pressure, though this was a consistently smaller effect, significant only in some studies [9,11,19,20,21]. A systematic review reporting CVD risk factors in children, including a meta-analysis of 18 published studies, showed that PE was associated with a 2.39 mm Hg higher systolic blood pressure [95% CI 1.74, 3.05) and 1.35 mmHg higher diastolic blood pressure [95% CI 0.90, 1.80) in children and adolescents (Davis et al., 2012) [10].

Attenuation of the blood pressure difference after adjusting for the children’s weight and height suggests that the higher blood pressure in children of PE mothers is at least partly mediated by the children’s increased body size. A similar attenuation has been reported previously [11,12,20]. Other mechanisms that lead to higher blood pressure in these children could include in utero programming of blood pressure regulation systems by exposure to preeclampsia, or shared (between mother and child) genes or family environment/behavioural factors. Factors/ exposures that may cause fetal programming include placental hypoxia, defective angiogenesis, endothelial dysfunction and immune modifications, possibly acting through epigenetic mechanisms, [22, 41, 42] and leading to hypertension through long-term changes in blood vessel development, kidney development or endocrine systems [19,35]. Our own earlier studies have reported altered maternal angiogenesis and differential placental expression and methylation of angiogenic factor genes in women with preeclampsia [27]. Alsnes et al compared blood pressure in adults from pregnancies complicated by hypertension with siblings from normotensive pregnancies and found no differences, favouring genetic or shared family environment mechanisms [9]. On the other hand, another sibling study (with a small sample size) showed vascular dysfunction (assessed using pulmonary artery pressure and flow-mediated brachial artery dilatation) among PE-exposed children but not among their siblings born from normal pregnancies [43], favouring an effect of the intra-uterine environment. The mechanisms of transmission of elevated blood pressure from mothers with PE to their children require further research.

### Sex differences

Some studies have reported significant increases in anthropometry or blood pressure only in boys [13,35,38]. We specifically explored this in our data, and found no evidence of sex differences.

### Strengths and limitations

As far as we know, ours is the largest cohort in India following up children born to women with and without preeclampsia. There was considerable attrition of the cohort since birth, either due to families moving away from their original address or because, due to lack of funds, we had to halt the study before we had time to re-contact all the families (Fig 1) and this could create bias. However, although there were statistically significant differences in maternal and neonatal characteristics between the children studied and not studied, these were small in absolute terms, and were similar between PE and non-PE groups, which make it unlikely that our finding of higher weight, height and skinfolds in PE children was due to selection bias. We had to suspend recruitment of women when the labour ward was very busy and the staff were under too much clinical pressure; however we think that this would have affected PE and non-PE women equally and would not therefore have introduced bias. Another limitation was that we did not check for a history of hypertension in previous pregnancies; if the effects of maternal preeclampsia on the children’s outcomes result from genetic or other familial causes (rather than the intrauterine environment), inclusion of mothers with a past history of preeclampsia or pregnancy induced hypertension as controls could dilute the observed effect size. Another limitation of the study is that we do not have consecutive anthropometric measurements across various ages in childhood which could have helped to understand the linear growth of children and at what age the PE children (who were born smaller) caught up and overtook the non-PE children. Also, we do not have information on the severity of the mother’s preeclampsia; some studies have shown greater differences in outcomes among children of women with severe preeclampsia.

## Conclusions

We have shown that, consistent with studies in high income countries, children born to mothers with preeclampsia were taller and heavier and had higher systolic blood pressure than children of non-PE mothers. These changes may indicate an increased risk of cardiovascular disease in adulthood. The mechanisms underlying these changes require further research.

## Supporting information

S1 Table Associations of child anthropometry and blood pressure with birthweight, gestational age, maternal BMI, maternal height, SLI score (socio-economic status), and the child’s age and sex. (DOC)

S2 Table Multiple regression analysis: Associations of maternal PE with anthropometric and blood pressure outcomes in the children, stratified by sex. (DOC)
