# Supplementary material for: Association of preeclampsia with anthropometric measures and blood pressure in Indian children
Source: PLoS One. 2020 May 5;15(5):e0231989. doi: 10.1371/journal.pone.0231989 (PMC7199948; doi:10.1371/journal.pone.0231989)
Supplement: S1 Table — (DOC) [file pone.0231989.s001.doc]

**Supplementary Table 1: Associations of child anthropometry and blood pressure with birthweight, gestational age, maternal BMI, maternal height, SLI score (socio-economic status), and the child’s age and sex**

|  | **Age of children** | | | **Sex** | | | **Birth weight** | | | **Gestational age** | | | **Maternal BMI** | | | **Maternal height** | | | **SLI score** | | |
| --- | --- | --- | --- | --- | --- | --- | --- | --- | --- | --- | --- | --- | --- | --- | --- | --- | --- | --- | --- | --- | --- |
|  | n | r | p | n | r | p | n | r | p | n | r | p | n | r | p | n | r | p | n | r | p |
| **z score of weight** |  |  |  |  |  |  | 674 | 0.33 | <0.001 | 674 | 0.10 | 0.01 | 517 | 0.25 | <0.001 | 642 | 0.23 | 0.001 | 620 | 0.22 | <0.001 |
| **z score of child** |  |  |  |  |  |  | 674 | 0.27 | <0.001 | 674 | 0.05 | 0.19 | 517 | 0.13 | 0.003 | 642 | 0.31 | 0.001 | 620 | 0.20 | <0.001 |
| **z score of BMI** |  |  |  |  |  |  | 674 | 0.26 | <0.001 | 674 | 0.10 | 0.007 | 517 | 0.27 | <0.001 | 642 | 0.31 | 0.12 | 620 | 0.15 | <0.001 |
|  |  |  |  |  |  |  |  |  |  |  |  |  |  |  |  |  |  |  |  |  |  |
| **MUAC (cm)** | 669 | 0.27 | <0.001 | 669 | -0.04 | 0.31 | 669 | 0.20 | <0.001 | 669 | 0.04 | 0.26 | 514 | 0.22 | <0.001 | 639 | 0.06 | 0.11 | 616 | 0.18 | <0.001 |
| **Head circumference (cm)** | 670 | 0.34 | <0.001 | 670 | -0.29 | 0.001 | 670 | 0.27 | <0.001 | 670 | 0.12 | 0.002 | 515 | 0.02 | 0.61 | 640 | 0.04 | 0.29 | 617 | 0.10 | 0.014 |
| **Chest circumference (cm)** | 670 | 0.40 | <0.001 | 670 | -0.26 | 0.001 | 670 | 0.19 | <0.001 | 670 | 0.01 | 0.85 | 515 | 0.16 | <0.001 | 640 | 0.03 | 0.49 | 617 | 0.10 | 0.013 |
| **Hip circumference (cm)** | 670 | 0.48 | <0.001 | 670 | -0.02 | 0.56 | 670 | 0.21 | <0.001 | 670 | 0.06 | 0.14 | 515 | 0.17 | <0.001 | 640 | 0.06 | 0.13 | 617 | 0.12 | 0.004 |
| **Waist circumference (cm)** | 499 | 0.32 | <0.001 | 499 | -0.10 | 0.03 | 499 | 0.20 | <0.001 | 499 | 0.02 | 0.60 | 435 | 0.19 | <0.001 | 471 | 0.02 | 0.68 | 447 | 0.11 | 0.018 |
| **Triceps (mm)** | 666 | 0.02 | 0.54 | 666 | 0.20 | 0.001 | 666 | 0.14 | <0.001 | 666 | 0.08 | 0.045 | 512 | 0.21 | <0.001 | 637 | 0.08 | 0.04 | 613 | 0.15 | <0.001 |
| **Biceps (mm)** | 668 | 0.03 | 0.45 | 668 | 0.18 | 0.001 | 668 | 0.10 | 0.01 | 668 | 0.03 | 0.47 | 513 | 0.15 | <0.001 | 638 | 0.08 | 0.04 | 615 | 0.18 | <0.001 |
| **Subscapular (mm)** | 665 | 0.06 | 0.14 | 665 | 0.15 | 0.001 | 665 | 0.07 | 0.07 | 665 | 0.02 | 0.59 | 511 | 0.18 | <0.001 | 636 | 0.05 | 0.22 | 612 | 0.15 | <0.001 |
| **Suprailiac (mm)** | 660 | -0.12 | <0.002 | 660 | 0.22 | 0.001 | 660 | 0.02 | 0.56 | 660 | -0.04 | 0.30 | 506 | 0.17 | <0.001 | 631 | 0.07 | 0.08 | 607 | 0.14 | <0.001 |
|  |  |  |  |  |  |  |  |  |  |  |  |  |  |  |  |  |  |  |  |  |  |
| **Systolic BP** | 614 | 0.26 | <0.001 | 614 | -0.07 | 0.09 | 614 | -0.01 | 0.84 | 614 | -0.06 | 0.12 | 466 | 0.06 | 0.20 | 587 | 0.02 | 0.71 | 593 | -0.01 | 0.74 |
| **Diastolic BP** | 614 | 0.04 | 0.354 | 614 | 0.13 | 0.002 | 614 | -0.01 | 0.72 | 614 | 0.01 | 0.79 | 466 | 0.05 | 0.26 | 587 | -0.01 | 0.90 | 593 | 0.00 | 0.94 |

BMI: body mass index, MUAC: mid-upper-arm circumference
