# Supplementary material for: Association of preeclampsia with anthropometric measures and blood pressure in Indian children
Source: PLoS One. 2020 May 5;15(5):e0231989. doi: 10.1371/journal.pone.0231989 (PMC7199948; doi:10.1371/journal.pone.0231989)
Supplement: S2 Table — (DOC) [file pone.0231989.s002.doc]

**Supplementary Table 2: Multiple regression analysis: Associations of maternal PE with anthropometric and blood pressure outcomes in the children, stratified by sex**

|  | **Unadjusted** | | | **Adjusted for birth weight and gestational age** | | | **Adjusted for birth weight, gestational age maternal BMI and SLI score** | | |
| --- | --- | --- | --- | --- | --- | --- | --- | --- | --- |
|  | n | Regression coefficient B (95% CI) | p | n | Regression coefficient B (95% CI) | p | n | Regression coefficient B (95% CI) | p |
|  | **Boys** | | | | | | | | |
| **Z score weight** | 373 | 0.31 (0.05, 0.56) | 0.02 | 373 | 0.50 (0.24, 0.76) | 0.001 | 263 | 0.40 (0.10, 0.69) | 0.009 |
| **Z score height** | 373 | 0.33 (0.10, 0.57) | 0.005 | 373 | 0.49 (0.26, 0.72) | 0.001 | 263 | 0.45 (0.19, 0.71) | 0.001 |
| **Z score BMI** | 373 | 0.10 (-0.16, 0.35) | 0.46 | 373 | 0.24 (-0.03, 0.51) | 0.08 | 263 | 0.13 (-0.19, 0.45) | 0.43 |
|  | **Girls** | | | | | | | | |
| **Z score weight** | 299 | 0.10 (-0.15, 0.35) | 0.45 | 299 | 0.32 (0.07, 0.57) | 0.01 | 203 | 0.25 (-0.06, 0.55) | 0.11 |
| **Z score height** | 299 | 0.12 (-0.11, 0.36) | 0.30 | 299 | 0.27 (0.03, 0.51) | 0.03 | 203 | 0.25 (-0.05, 0.55) | 0.10 |
| **Z score BMI** | 299 | 0.03 (-0.22, 0.27) | 0.82 | 299 | 0.22 (-0.02, 0.46) | 0.08 | 203 | 0.13 (-0.18, 0.44) | 0.41 |
|  | **Adjusted for child age** | | | **Adjusted for child age, birth weight and gestational age at birth** | | | **Adjusted for child age, birth weight, gestational age and maternal BMI and SLI score** | | |
|  | n | Regression coefficient B (95% CI) | p | n | Regression coefficient B (95% CI) | p | n | Regression coefficient B (95% CI) | p |
|  | **Boys** | | | | | | | | |
| **Anthropometry** |  |  |  |  |  |  |  |  |  |
| **MUAC (cm)** | 370 | 0.26 (-0.07, 0.59) | 0.13 | 370 | 0.42 (0.08, 0.77) | 0.02 | 261 | 0.22 (-0.18, 0.61) | 0.28 |
| **Head circumference(cm)** | 371 | -0.01 (-0.32, 0.31) | 0.96 | 371 | 0.24 (-0.08, 0.55) | 0.14 | 262 | 0.19 (-0.19, 0.57) | 0.32 |
| **Triceps skinfold (mm)** | 369 | 0.35 (-0.07, 0.77) | 0.11 | 369 | 0.55 (0.11, 0.99) | 0.01 | 261 | 0.48 (-0.07, 1.02) | 0.09 |
| **Biceps skinfold (mm)** | 371 | 0.29 (-0.08, 0.66) | 0.12 | 371 | 0.41 (0.03, 0.80) | 0.04 | 262 | 0.33 (-0.16, 0.82) | 0.18 |
| **Subscapular skinfold (mm)** | 368 | 0.45 (0.05, 0.86) | 0.03 | 368 | 0.56 (0.13, 0.99) | 0.01 | 260 | 0.41 (-0.14, 0.96) | 0.14 |
| **Suprailiac skinfold (mm)** | 366 | 0.51 (-0.18, 1.21) | 0.15 | 366 | 0.44 (-0.29, 1.18) | 0.25 | 258 | 0.48 (-0.41, 1.36) | 0.29 |
| **Blood pressure** |  |  |  |  |  |  |  |  |  |
| **Systolic BP (mmHg)** | 346 | 1.74 (0.19, 3.29) | 0.03 | 346 | 2.02 (0.40, 3.65) | 0.02 | 191 | 1.78 (-0.19, 3.75) | 0.08 |
| **Diastolic BP (mmHg)** | 346 | 0.90 (-0.76, 2.56) | 0.29 | 346 | 1.15 (-0.60, 2.90) | 0.20 | 254 | 1.34 (-0.81, 3.49) | 0.22 |
|  | **Girls** | | | | | | | | |
| **MUAC (cm)** | 297 | 0.11 (-0.22, 0.44) | 0.52 | 297 | 0.34 (0.05, 0.67) | 0.045 | 202 | 0.28 (-0.14, 0.70) | 0.19 |
| **Head circumference(cm)** | 297 | -0.28 (-0.60, 0.05) | 0.09 | 297 | -0.02 (-0.35, 0.32) | 0.93 | 202 | 0.0(-0.37, 0.48) | 0.80 |
| **Triceps skinfold (mm)** | 295 | -0.04 (-0.52, 0.43) | 0.86 | 295 | 0.19 (-0.31, 0.69) | 0.46 | 200 | -0.11 (-0.74, 0.52) | 0.74 |
| **Biceps skinfold (mm)** | 295 | 0.13 (-0.22, 0.50) | 0.47 | 295 | 0.26 (-0.12, 0.63) | 0.18 | 200 | 0.10 (-0.35, 0.54) | 0.67 |
| **Subscapular skinfold (mm)** | 295 | 0.16 (-0.22, 0.55) | 0.41 | 295 | 0.30 (-0.11, 0.71) | 0.15 | 200 | 0.37 (-0.15, 0.88) | 0.16 |
| **Suprailiac skinfold (mm)** | 292 | 0.39 (-0.36, 1.14) | 0.30 | 295 | 0.50 (-0.30, 1.29) | 0.22 | 197 | 0.52 (-0.49, 1.53) | 0.31 |
| **Blood pressure** |  |  |  |  |  |  |  |  |  |
| **Systolic BP (mmHg)** | 266 | 1.61 (-0.29, 3.50) | 0.10 | 266 | 1.31 (-0.70, 3.32) | 0.20 | 191 | 1.5 (-1.22, 4.14) | 0.28 |
| **Diastolic BP (mmHg)** | 266 | 0.11 (-1.63, 1.86) | 0.90 | 266 | 0.09 (-1.76, 1.95) | 0.92 | 191 | 0.11 (-2.29, 2.51) | 0.93 |

BMI: body mass index, MUAC: mid-upper-arm circumference
